# How the Wnt signaling pathway protects from neurodegeneration: the mitochondrial scenario

**DOI:** 10.3389/fncel.2015.00166

**Published:** 2015-05-05

**Authors:** Macarena S. Arrázola, Carmen Silva-Alvarez, Nibaldo C. Inestrosa

**Affiliations:** ^1^Facultad de Ciencias Biológicas, Departamento de Biología Celular y Molecular, Centro de Envejecimiento y Regeneración (CARE), Pontificia Universidad Católica de ChileSantiago, Chile; ^2^Center for Healthy Brain Aging, School of Psychiatry, Faculty of Medicine, University of New South WalesSydney, NSW, Australia; ^3^Centro de Excelencia en Biomedicina de Magallanes (CEBIMA), Universidad de MagallanesPunta Arenas, Chile; ^4^Centro UC Síndrome de Down, Pontificia Universidad Católica de ChileSantiago, Chile

**Keywords:** Wnt, mitochondrial dynamics, permeability transition, Alzheimer’s disease, amyloid-beta, Drp1, electron microscopy

## Abstract

Alzheimer’s disease (AD) is the most common neurodegenerative disorder and is characterized by progressive memory loss and cognitive decline. One of the hallmarks of AD is the overproduction of amyloid-beta aggregates that range from the toxic soluble oligomer (Aβo) form to extracellular accumulations in the brain. Growing evidence indicates that mitochondrial dysfunction is a common feature of neurodegenerative diseases and is observed at an early stage in the pathogenesis of AD. Reports indicate that mitochondrial structure and function are affected by Aβo and can trigger neuronal cell death. Mitochondria are highly dynamic organelles, and the balance between their fusion and fission processes is essential for neuronal function. Interestingly, in AD, the process known as “mitochondrial dynamics” is also impaired by Aβo. On the other hand, the activation of the Wnt signaling pathway has an essential role in synaptic maintenance and neuronal functions, and its deregulation has also been implicated in AD. We have demonstrated that canonical Wnt signaling, through the Wnt3a ligand, prevents the permeabilization of mitochondrial membranes through the inhibition of the mitochondrial permeability transition pore (mPTP), induced by Aβo. In addition, we showed that non-canonical Wnt signaling, through the Wnt5a ligand, protects mitochondria from fission-fusion alterations in AD. These results suggest new approaches by which different Wnt signaling pathways protect neurons in AD, and support the idea that mitochondria have become potential therapeutic targets for the treatment of neurodegenerative disorders. Here we discuss the neuroprotective role of the canonical and non-canonical Wnt signaling pathways in AD and their differential modulation of mitochondrial processes, associated with mitochondrial dysfunction and neurodegeneration.

## Introduction

Mitochondria are key organelles for proper neuronal function and viability, not only for their role in ATP production but also for their tremendous capacity to buffer intracellular calcium (Ca^2+^; Celsi et al., 2009). Due to this important function in maintaining neuronal Ca^2+^ homeostasis, mitochondria have been indicated in the regulation of synaptic transmission (Billups and Forsythe, 2002). To perform this function, mitochondria need to be mobile and to divide and fuse in order to enter compartments where they are required for energy. These include growth cones, axonal branches, presynaptic terminals and dendritic spines (Morris and Hollenbeck, 1993; Ruthel and Hollenbeck, 2003; Li et al., 2004). Since mitochondria are involved in several essential processes of neuronal function, pathological changes in mitochondrial dynamics, traffic or in their structure directly affect mitochondrial function, not only by failing to produce ATP and to buffer Ca^2+^ but also by producing apoptotic cell death signals and contributing to neurodegenerative diseases (Detmer and Chan, 2007; Su et al., 2010; Sheng and Cai, 2012), such as Alzheimer’s disease (AD; Eckert et al., 2003; Newmeyer and Ferguson-Miller, 2003). AD is one of the most common causes of dementia worldwide and mitochondrial dysfunction has been widely associated with degeneration observed during the early stages of the disease (Du et al., 2010; Swerdlow et al., 2010; Balietti et al., 2013). For this reason we are interested in studying how we can protect mitochondria to prevent the neuronal dysfunction observed in AD. Moreover, one of the most important signaling cascades described as being neuroprotective against amyloid-β peptide (Aβ) in AD is the Wnt signaling pathway (De Ferrari and Inestrosa, 2000; Cerpa et al., 2009; Inestrosa and Varela-Nallar, 2014), which has been deeply implicated in the development and maintenance of the nervous system (Salinas and Zou, 2008; Inestrosa and Arenas, 2010; Rosso and Inestrosa, 2013). Furthermore, Wnt signaling is differentially activated in neurons to exert pre- or post-synaptic protective effects (Chacón et al., 2008; Cerpa et al., 2010). Here, we propose to describe the effects of canonical and non-canonical Wnt signaling activation as a novel mechanism to protect mitochondria from common defects associated with the pathogenesis of AD, such as impaired mitochondrial dynamics, loss of calcium buffering capacity, disruption of mitochondrial membranes through the induction of the mitochondrial permeability transition pore (mPTP) and the loss of their structure and function that finally produce neuronal cell death in AD.

## Alzheimer’s Disease

AD is one of the most common neurodegenerative disorders, characterized by a progressive loss of memory and cognitive decline (Hardy and Selkoe, 2002). At the neuropathological level, brains of AD patients are characterized by the presence of senile plaques, which are extracellular depositions of Aβ aggregates (Mattson, 2004). Although it is not clearly known which is the molecular triggering factor of the disease in most AD patients, there are several studies suggesting that the Aβ peptide plays a key pathogenic role (Hardy and Selkoe, 2002; Bates et al., 2009). Aβ peptide is generated by the proteolytic processing of the amyloid precursor protein (APP; Chow et al., 2010) and once it has been produced, it can aggregate to form soluble species known as Aβ oligomers (Aβo) and insoluble aggregates called amyloid fibrils, which are the result of a higher state of aggregation (Morgan et al., 2004; Ross and Poirier, 2005). Both aggregates form senile plaques in AD brains (Sakono and Zako, 2010). Despite the fact that Aβ fibrils are considered as the neurotoxic species that apparently triggers AD (Hardy and Higgins, 1992; Morgan et al., 2004) both *in vivo* and *in vitro* (Alvarez et al., 2004; Dinamarca et al., 2006), currently, there is consensus that Aβo could be the main cause of AD neurotoxicity, since they are the species responsible for the synaptic dysfunction observed in this pathology (Walsh et al., 2002; Cerpa et al., 2008; Li et al., 2009). Recent studies have shown a strong correlation between Aβo levels and the severity of synaptic and cognitive damage (McLean et al., 1999; Ferreira et al., 2007; Haass and Selkoe, 2007), suggesting that Aβo are the main effectors of synaptic loss and neuronal degeneration in AD (Lambert et al., 1998; Cleary et al., 2005; Cerpa et al., 2008).

Besides synapses, Aβ presents several molecular and cellular targets in neurons, contributing to the neuronal damage that this peptide generates in AD. One of these targets is Ca^2+^, which is a fundamental ion in the physiology of neurons, since it modulates many neuronal processes, including membrane excitability, neurotransmitter release, gene expression, and neuronal growth and viability (LaFerla, 2002; Bezprozvanny and Mattson, 2008). The balance between ion influx and release modulates Ca^2+^ signaling. Ca^2+^ influx across the plasma membrane occurs through voltage-gated Ca^2+^ channels, NMDA receptors and transient receptor potential channels (Alford et al., 1993; Berridge, 1998). Ca^2+^ release from intracellular Ca^2+^ stores occurs via inositol triphosphate receptor (IP_3_R) and ryanodine receptor (RyR) channels in the endoplasmic reticulum (ER; Marks, 1997). Moreover, mitochondria also participate in the regulation of neuronal Ca^2+^ levels through Ca^2+^ uptake, stimulating mitochondrial metabolism and energy production (Babcock et al., 1997). However, excessive calcium uptake into mitochondria can lead to the opening of the permeability transition pore (PTP) and, subsequently, to apoptosis (Spät et al., 2008; Celsi et al., 2009).

During the slow progression of AD, the early phase of memory loss is exacerbated by the onset of neuronal death, which may also be driven by an increased deregulation of Ca^2+^ homeostasis (Demuro et al., 2010; Berridge, 2011). It has been proposed that Aβ interaction with the plasma membrane results in elevated intracellular Ca^2+^ concentrations and increased vulnerability of neurons to excitotoxicity (Mattson et al., 1992). The oligomeric forms of Aβ may increase Ca^2+^ entry by either functioning as channels or by activating channels at the plasma membrane such as the NMDA receptor (Dinamarca et al., 2010; Berridge, 2011). The increase in cytosolic Ca^2+^ directly affects mitochondria, disrupting their critical function as Ca^2+^ buffering organelles (Celsi et al., 2009), which disturbs ATP generation and therefore neuronal viability. For this reason, mitochondrial dysfunction appears an obligatory downstream step in the pathogenesis of AD.

## Mitochondrial Deregulation in AD

Mitochondrial dysfunction is an early feature of AD since several abnormalities have been described in brains from different AD models (Moreira et al., 2007; Supnet and Bezprozvanny, 2010; Swerdlow et al., 2010). The activity of respiratory chain enzymes associated with the mitochondrial complex III (cytochrome-c reductase) and IV (cytochrome-c oxidase) is significantly decreased in mitochondria from transgenic (Tg) APP mice (Caspersen et al., 2005; Manczak et al., 2006) and in isolated mitochondria exposed to Aβ *in vitro* (Canevari et al., 1999). Generation of reactive oxygen species (ROS) is also deregulated in AD and an enhanced production of free radicals and oxidative damage is a feature of the progression of the disease (Reddy, 2006). In addition, metabolic properties, such as ATP levels and glucose uptake are also decreased in AD brains from Tg APP mice (Mosconi, 2005; Gauthier et al., 2006; Yao et al., 2009; Chen and Yan, 2010). The mitochondrial alterations triggered by Aβ creates a negative environment for the maintenance of mitochondrial function, since it directly affects the electrochemical gradient that is generated along the electron transport chain (ETC), favoring electron leakage and the production of superoxide species, loss of mitochondrial membrane potential (mΔΨ), permeability and structure disruption (Reddy, 2009). It has been clearly demonstrated that Aβ accumulates progressively within AD brains, Tg mouse models and cells overexpressing APP (Lustbader et al., 2004; Devi et al., 2006; Du et al., 2008). Aβ produced in the extracellular space enters neurons via the endocytic pathway (Yu et al., 2010) and once amyloid-beta oligomers (Aβo) are localized inside neurons, these oligomers directly affect mitochondria. Mitochondrial accumulation of Aβ could explain why Aβ dramatically interferes with the function and structure of this organelle, thus affecting neuronal viability.

Aβ accumulation in mitochondria occurs early in brains of Tg APP mice, between 4–5 months old, and increases with age, even before the massive extracellular deposition occurs (Caspersen et al., 2005); an observation in agreement with previous findings that indicate that intracellular accumulation of Aβ occurs previously to amyloid plaque formation (Wirths et al., 2001). The first study that described the presence of Aβ in the mitochondria was by Lustbader et al. (2004). They demonstrated that Aβ co-localized with the Aβ-binding alcohol dehydrogenase (ABAD) inside mitochondria from human AD brains, and that the interaction between Aβ and ABAD promotes leakage of ROS, mitochondrial dysfunction and cell death (Lustbader et al., 2004). It has been proposed that APP could be located at the outer mitochondrial membrane (OMM) where it can be processed by mitochondrial γ-secretase (Devi and Anandatheerthavarada, 2010). The import of Aβ into mitochondria has been observed both *in vivo* and *in vitro* and occurs through the mitochondrial protein transport machinery, specifically via the translocase of the OMM (TOM). This phenomenon has been observed even when the Aβ peptide is extracellularly added (Hansson Petersen et al., 2008), therefore, mitochondrial Aβ accumulation is a key process, which leads to the understanding of how Aβ can damage neurons so effectively in AD. Once inside mitochondria, Aβ can interact with several proteins that are important for the correct function of this organelle and/or for the maintenance of its structure, such as cyclophilin D (CypD), which participates in the opening of the mPTP and therefore regulates the permeability and function of mitochondria, affecting both their energetic and calcium buffering functions (Connern and Halestrap, 1994; Du and Yan, 2010b).

The mPTP is a non-selective pore that remains open for periods that are highly dependent on calcium concentration inside the mitochondrial matrix (MM). Opening of the mPTP for short time periods induces a rapid and regulated calcium release from the MM (Halestrap, 2009). If the pore remains open for longer periods of time, potentiated by an apoptotic stimulus, such as Aβ, or by elevated calcium concentrations, an uncontrolled release of this ion from the mitochondria occurs (Muirhead et al., 2010; Rao et al., 2014). As a result, the permeabilization of the inner mitochondrial membrane (IMM) induces morphological changes in the mitochondria, including increased volume, a phenomenon known as *swelling*, and the dissipation of the mΔΨ, disruption of membranes and uncontrolled release of calcium and pro-apoptotic factors, such as cytochrome-c, into the cytoplasm, activating neuronal cell death cascades (Petronilli et al., 2001). Thus, blocking Aβ action in mitochondria, and therefore its ability to induce mPTP opening, are potential therapeutic strategies for AD. For this reason, the study of molecules that could prevent mitochondrial permeability induced by Aβ is crucial for the development of tools for the early treatment of neurodegenerative diseases in which mitochondria are involved.

## Mitochondrial Dynamics in Neurodegenerative Diseases

The mitochondrion is a highly dynamic organelle that can migrate, split and merge. The regulation of these processes is critical for normal cell function. Mitochondrial morphology is very heterogeneous and may vary from small spheres to interconnected tubules. These structural fluctuations are regulated by the mitochondrial fission-fusion process, also known as mitochondrial dynamics, which controls several mitochondrial qualities and features, such as energy metabolism, mitochondrial DNA content, and the shape and number of mitochondria inside the cell (Hoppins et al., 2007). In addition to these primary functions, several reports have demonstrated that these dynamic processes are critical for the regulation of cell death, mitophagy, and organelle distribution (Itoh et al., 2013; Kornmann, 2014). A disruption in the balance between mitochondrial fission and fusion processes is associated with neurodegenerative diseases, such as AD, Parkinson’s disease (PD) and Charcot-Mary-Tooth (CMT) disease type 2A (Detmer and Chan, 2007; Schon and Przedborski, 2011; Yoon et al., 2011; Burté et al., 2015).

Mitochondrial fission is regulated by dynamin-related protein 1, Drp1 (Kageyama et al., 2011; Tamura et al., 2011), which is a cytosolic protein that assembles around mitochondria to constrict and split them (Labrousse et al., 1999; Smirnova et al., 2001). Drp1 is physiologically relevant during the embryonic development of humans. A Drp1 mutation (A395D) that causes an impaired assembly of Drp1 at mitochondria, leading to decreased fission, elongated mitochondria, and their altered cellular distribution, was correlated with microcephaly, abnormal development of the brain and had lethal consequences in a newborn patient (Chang et al., 2010).

In mammals, the mechanism of Drp1 translocation to mitochondria is regulated by calcineurin-dependent dephosphorylation (Cereghetti et al., 2008). Numerous regulatory posttranslational modifications of Drp1 have been reported including phosphorylation by PKC-δ (Qi et al., 2011) and Cdk (Cdk1)/cyclin-B (Taguchi et al., 2007) and nitrosylation (Barsoum et al., 2006). Ubiquitination and sumoylation of Drp1 also regulate its stability and its interaction with mitochondria (Chang and Blackstone, 2010; Wilson et al., 2013). These modifications are essential for controlling mitochondrial dynamics, since the deregulation in the phosphorylation-dephosphorylation balance of Drp1 leads to the loss of mitochondrial homeostasis and apoptosis (Cribbs and Strack, 2007; Hoppins et al., 2007).

By contrast, mitochondrial fusion is regulated by two proteins widely expressed in the OMM of the brain and other tissues, known as mitofusin (Mfn) 1 and 2 (Rojo et al., 2002; Eura et al., 2003). During this process, Mfn1 and 2 couple two adjacent mitochondria to fuse both OMMs (Koshiba et al., 2004), while the IMM fusion is mediated by the Opa1 protein (optic atrophy 1), which interacts with Mfns to fuse both membranes (Cipolat et al., 2004; Song et al., 2009). Pathological mutations in Mfn2 and Opa1 impede mitochondrial fusion, where Mfn2 causes the axonal CMT disease type 2A, which is characterized by the degeneration of retinal ganglion cells and the optic nerve, and Opa1 causes autosomal dominant optic atrophy, with the degeneration of peripheral sensory and motor neurons (Westermann, 2010; Burté et al., 2015).

In AD, Aβ overproduction causes an imbalance in mitochondrial dynamics, affecting the levels of Drp1, Opa1, Mfn1 and 2, both *in vitro* and in postmortem brains of AD patients (Wang et al., 2008, 2009), inducing mitochondrial fragmentation and an abnormal distribution of mitochondria inside neurons. Despite the fact that the mechanism of deregulation of mitochondrial dynamics in AD is not completely understood, it is well known that the activation state of Drp1 seems to be relevant (Cho et al., 2009). Another possible mechanism, which also involves Drp1, is the increased expression and interaction of Drp1 with Aβ and phosphorylated tau observed in AD patients (Manczak et al., 2011; Manczak and Reddy, 2012). However, the controversial evidence of Drp1 levels involved in AD needs to be determined. Moreover, in brains from Tg2576 animals at the early stage of the disease, a reduced number of synapses was observed, containing a higher number of mitochondria, which were smaller in size (Balietti et al., 2013), indicating an early vulnerability of this region to the damage induced by Aβ. So, changes in mitochondrial dynamics suggest a compensatory plastic mechanism to respond against new energetic requirements that are necessary for synaptic remodeling.

Besides AD, the dysfunctions of mitochondrial dynamics are also a hallmark in the pathogenesis of several neurodegenerative diseases such as PD. PD occurs mainly as a sporadic disease, which is characterized by the intracellular accumulation of α-synuclein (SNCA) protein. A lower proportion of PD is produced by inherited genetic mutations, and its onset occurs earlier than sporadic PD. Among the genes altered in PD and that are associated with mitochondrial dysfunction, are found *α-synuclein*, and *leucine-rich repeat kinase 2* (LRRK2), which are linked to autosomal-dominant PD. By contrast, *parkin* and *PTEN-induced putative kinase 1* (PINK) mutations are linked to autosomal-recessive parkisonisms (Alberio et al., 2012; Blandini and Armentero, 2012). Although no studies have clearly established the causes of this disease, it has been proposed that the overexpression of SNCA indirectly induces mitochondrial fragmentation in neurons from a PD Tg mouse model *in vivo*, by a mechanism independent to the fission-fusion machinery (Xie and Chung, 2012). LRRK2 mutations are the most common cause of familial and autosomal-dominant PD. LRRK2 interacts with Drp1, inducing mitochondrial fragmentation, which is increased in the presence of PD-associated mutations, both *in vitro* and *in vivo* (Wang et al., 2012; Burté et al., 2015). The PINK/Parkin pathway is known to regulate homeostasis and mitochondrial quality control. Studies in fibroblasts from patients with PD showed that Parkin promotes mitochondrial elongation by degradation of Drp1 through its ubiquitination (Yan et al., 2013). However more studies should be performed to clarify the discrepancies of these results obtained between Drosophila and mammals (Exner et al., 2007; Wang et al., 2011). This evidence shows a strong relationship between disturbed mitochondrial dynamics and neurodegeneration.

## The Role of Wnt Signaling in Neuroprotection

Previous studies from our laboratory have demonstrated that the activation of the canonical Wnt signaling pathway protects neurons against Aβ toxicity *in vitro* and *in vivo* (Alvarez et al., 2004; Toledo and Inestrosa, 2010; Silva-Alvarez et al., 2013; Vargas et al., 2014). The Wnt ligands are secreted proteins that mainly activate two Wnt pathways: the canonical, or β-catenin-dependent signaling pathway (Wnt/β-catenin); and the non-canonical signaling pathways (Wnt/PCP and Wnt/Ca^2+^) (Willert and Nusse, 2012; Inestrosa and Varela-Nallar, 2014). The Wnt/PCP pathway is activated by the interaction of a Wnt ligand with its Frizzled (Fz) receptor. This binding produces the activation of Disheveled (Dvl), which in turn activates the small GTPases Rho and Rac, to finally activate the Jun N-terminal kinase (JNK), regulating cytoskeleton reorganization (Rosso et al., 2005). On the other hand, in the Wnt/Ca^2+^ pathway, the binding between a Wnt ligand and the Fz receptor activates the trimeric G proteins, which induce the activation of the phospholipase C (PLC) and the production of diacylglycerol (DAG) and inositol triphosphate (IP3), generating an increase in intracellular Ca^2+^ levels and the subsequent activation of Ca^2+^-dependent proteins (Inestrosa and Arenas, 2010). By contrast, the Wnt/β-catenin pathway is activated by the binding of a Wnt ligand to its Fz receptor and to the co-receptor LRP5/6 (Willert and Nusse, 2012; Liu et al., 2014). This interaction activates Dvl and causes the dissociation of the destruction complex to inhibit the glycogen synthase kinase-3β (GSK-3β) and to prevent β-catenin degradation through the proteasome, inducing its accumulation into the cytoplasm, which finally translocates to the nucleus and triggers the expression of Wnt target genes (Arrázola et al., 2009; Clevers and Nusse, 2012). Canonical Wnt signaling has been implicated in neuroprotection against Aβ-induced neuronal damage (Cerpa et al., 2009). In fact, its activation protects hippocampal neurons from Aβ-induced cell death (Alvarez et al., 2004), and also prevents the intracellular calcium increase generated by Aβ in neurons, which directly affects mitochondrial calcium levels (Quintanilla et al., 2005; Dinamarca et al., 2010). In addition, we have demonstrated that the non-canonical Wnt pathway, through its ligand Wnt5a, also has a neuroprotective response against Aβ exposition through the modulation of mitochondrial dynamics (Silva-Alvarez et al., 2013). However, the mechanism by which this protection occurs is unknown. Recently, our lab explored whether Wnt signaling could exert its neuroprotective role against Aβ-induced toxicity through the protection of the mitochondria (Arrázola and Inestrosa, 2013).

## Protective Effects of the Different Wnt Pathways

### Canonical: Protects Mitochondria from Permeability Transition

Wnt/β-catenin signaling activation has been involved in the development and maintenance of the nervous system (Inestrosa and Arenas, 2010; Inestrosa and Varela-Nallar, 2015) because it regulates synaptogenesis (Salinas and Zou, 2008; Rosso and Inestrosa, 2013) and participates in adult neurogenesis of the hippocampus (Lie et al., 2005; Varela-Nallar and Inestrosa, 2013). Besides these critical functions in the physiology of the central nervous system, the canonical Wnt pathway has been associated with cell survival and neuroprotection (Oliva et al., 2013; Harvey and Marchetti, 2014). For several years, our laboratory has been studying the role of Wnt signaling in the neurodegeneration observed in AD. The first time that canonical Wnt signaling was associated with AD was by De Ferrari and Inestrosa in 2000, where they proposed that sustained loss of Wnt signaling function may lead to AD (De Ferrari and Inestrosa, 2000; Inestrosa and Varela-Nallar, 2014). Later, they showed that Aβ fibrils induced the destabilization of endogenous levels of β-catenin, which were recovered with lithium (De Ferrari et al., 2003), a pharmacological inhibitor of GSK-3β, and therefore an inductor of Wnt signaling activation (Klein and Melton, 1996). In addition, our laboratory demonstrated that direct activation of the canonical Wnt signaling pathway, through the Wnt3a ligand, prevents neuronal death induced by Aβ fibrils and rescues β-catenin levels (Alvarez et al., 2004), indicating that Wnt signaling activation plays a key role in neuroprotection against Aβ-induced neuronal cell death (Cerpa et al., 2009).

It is very well established that calcium plays an important role in the neurotoxicity of AD in mouse models and *in vitro* (LaFerla, 2002; Stutzmann et al., 2007), as it has been demonstrated that Aβ aggregates, prepared from Aβ synthetic peptides, induce intracellular calcium increase, which directly impacts mitochondrial calcium levels (Dinamarca et al., 2010; Supnet and Bezprozvanny, 2010). Previous studies with Wnt7a indicated that this ligand prevents the intracellular calcium increase generated by Aβ fibrils (Quintanilla et al., 2005) and Aβ oligomers (Dinamarca et al., 2010) in hippocampal neurons. These oligomers affect the calcium buffering function of the mitochondria, as they produce calcium overload into the organelle, however it has not been explored whether Wnt signaling is able to prevent the calcium entrance into mitochondria and if this process is involved in the neuroprotective role of this signaling pathway against Aβ toxicity in AD.

Regarding the calcium buffering function of the mitochondria, it is well known that a massive and uncontrolled calcium influx into mitochondria directly affects their permeability (Du and Yan, 2010a) through mPTP opening (Szalai et al., 1999). When the IMM is disrupted, because of the formation of the mPTP, mitochondria undergo some structural changes that finally affect its function and therefore, the cellular decision between life or death (Newmeyer and Ferguson-Miller, 2003). The loss of mΔΨ and the release of mitochondrial proapototic factors are events triggered by mitochondrial *swelling*, which occurs during mPTP formation and that enables the exchange of solutes between the cytoplasmic compartment and MM, and viceversa, generating increased volume of the organelle, membrane disruption and, finally, the loss of mitochondrial function and cell death (Petronilli et al., 2001). All of these events have been described in AD as a consequence of the mitochondrial permeability transition (Moreira et al., 2001) induced by Aβ-mediated calcium variations (Celsi et al., 2009) or through a direct interaction of Aβ with the mPTP protein complex (Du and Yan, 2010b).

**Figure 1:**
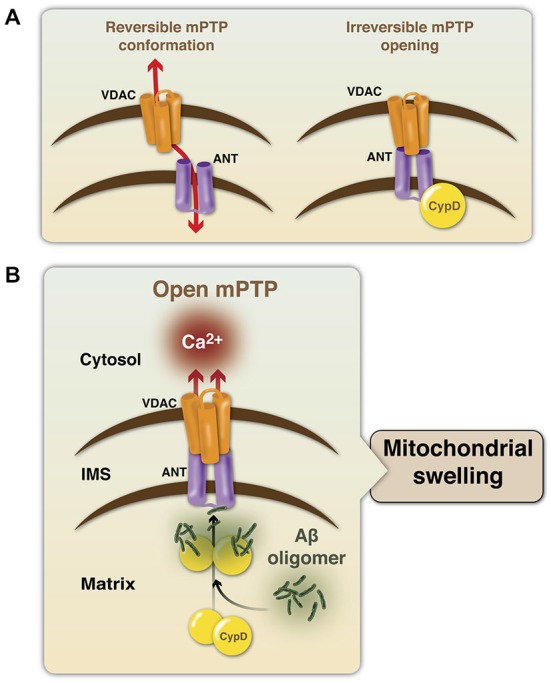
**Modulation of the mitochondrial permeability transition pore structure. (A)** The mPTP is mainly formed by the voltage-dependent calcium channel (VDAC, in orange), adenine nucleotide translocase (ANT, in purple) and cyclophilin D (CypD, in yellow). Under a physiological state, the mPTP presents a reversible conformation and CypD is not part of the protein complex and mainly resides into the mitochondrial matrix (MM). An interaction of the components, triggered by an apoptotic stimulus, induces the formation and opening of the mPTP, generating an irreversible response, that allows the entry of water and solutes into the MM. **(B)** In the context of AD, Aβ oligomers induce the translocation of CypD from the MM to the IMM to facilitate their interaction with ANT, and therefore the formation and opening of the mPTP. This final step induces uncontrolled release of calcium and mitochondrial swelling.

The mPTP is mainly formed by three proteins: the voltage-dependent anion channel (VDAC) at the OMM, adenine nucleotide translocase (ANT) at the IMM, and CypD in the MM; Baines et al., 2005; Schinzel et al., 2005; Halestrap, 2009). VDAC is known as the mitochondrial porin and regulates cell life and death, as it controls the entry an exit of mitochondrial metabolites (Shoshan-Barmatz et al., 2010). ANT is an ADP/ATP transporter, but is known to switch its function to a pore-forming channel during apoptosis, regulating mPTP formation (Tsujimoto and Shimizu, 2007; Singh et al., 2009). CypD is the mitochondrial isoform of the peptidylprolyl cis- trans isomerase cyclophilin chaperone family (Halestrap et al., 1997; Crompton et al., 1998). CypD is the most important initiating molecule for the mPTP, as it is a positive regulator required for mPTP formation (Schinzel et al., 2005). CypD translocates to the IMM during the opening of the pore under oxidative stress conditions, interacting with ANT (Connern and Halestrap, 1994) and inducing the irreversible conformation of the mPTP (Figure 1A). Regarding the action of Aβ on mPTP formation, it is known that Aβ directly interacts with CypD, favoring the formation of the pore, mitochondrial swelling and the release of calcium from the mitochondria (Figure 1B; Du et al., 2008). Thus, blocking the action of Aβ in mitochondria and mPTP induction could be potential therapeutic strategies against AD.

Recently, our laboratory showed the effect of the canonical Wnt signaling activation on mPTP formation induced by Aβo. Live cell imaging assays performed to evaluate mPTP induction *in situ* in cultured hippocampal neurons (Gillessen et al., 2002; Arrázola and Inestrosa, 2015) indicated that the canonical ligand Wnt3a inhibits mPTP opening, induced by Aβo. We also showed by electron microscopy from hippocampal slices that, besides mPTP inhibition mediated by Wnt3a, this ligand can also prevent the disruption of the mitochondrial membranes and mitochondrial swelling, since the volume and size of the organelle were conserved when Wnt signaling was activated, preserving the whole structure of the mitochondria even in the presence of Aβo (Arrázola and Inestrosa, 2013), and preventing the changes that occur in AD brains during mPTP opening (Moreira et al., 2001, 2002; Du and Yan, 2010b; Du et al., 2010).

Pharmacological studies using ICG-001, an inhibitor of Wnt-dependent gene transcription (Emami et al., 2004), allowed us to determine that mPTP inhibition mediated by Wnt3a was a β-catenin-independent effect (Arrázola and Inestrosa, 2013), indicating that Wnt modulates mPTP opening through a mechanism that involves a divergent canonical Wnt signaling pathway. According to this idea, it has been reported that GSK-3β, one of the main components of the canonical Wnt signaling pathway, is able to translocate to the mitochondria and to interact with ANT from the mPTP complex (Nishihara et al., 2007; Gomez et al., 2008; Juhaszova et al., 2009). This interaction has been described between ANT and the inhibited form of GSK-3β (phosphorylated at serine 9), and it correlates with a decrease in the CypD-ANT interaction, which is necessary for mPTP opening (Miura et al., 2009; Zorov et al., 2009; Miura and Tanno, 2010). Interestingly, canonical Wnt signaling activation involves the phosphorylation of cytoplasmic GSK-3β at the same residue (Ser-9), inhibiting the kinase (Stambolic and Woodgett, 1994) and surprisingly inducing the levels of phosphorylated-GSK-3β at the mitochondria. Moreover, we observed that this mitochondrial pool of phosphorylated-GSK-3β specifically interacts with ANT and not with CypD, when Wnt signaling is activated (Arrázola and Inestrosa, 2013), supporting the idea that the mechanism by which Wnt3a inhibits mPTP opening could be mediated by the modulation of mitochondrial GSK-3β; as has been recently described in different models of mPTP induction (Juhaszova et al., 2004; Nishihara et al., 2007; Miura and Tanno, 2010; Tanno et al., 2014).

On the other hand, several proteins have been described as regulators of mPTP formation and all of them can interact with some of the main components of the pore, or affect or facilitate their interaction in order to modulate mitochondrial membrane permeability and cell viability (Eliseev et al., 2009; Rasola et al., 2010a; Saraiva et al., 2010). An example is the mitochondrial hexokinase II (HKII) that when detached from the mitochondria it induces mPTP formation and cell death (Chiara et al., 2008). Mitochondrial HKII regulates mPTP induction through the delivery of a survival signal that stabilizes the mPTP in the closed conformation, whereas HKII detachment from mitochondria would propagate a conformational change to molecules of the inner mitochondrial membrane, eventually leading to pore opening (Rasola et al., 2010b). Another mPTP modulator is the survival kinase Akt, which regulates HKII by promoting its binding to mitochondria, through HKII phosphorylation (Miyamoto et al., 2008). Interestingly and in agreement with our studies, Akt also inactivates GSK-3β by phosphorylation, a fact that has been used in favor of the association of HKII to the OMM, and therefore to the inhibition of mPTP opening. By contrast, activation of GSK-3β was shown to release HKII, enhancing susceptibility to cell death (Robey and Hay, 2006). Interestingly in AD, GSK-3β interacts with VDAC (Nishihara et al., 2007), to dissociate HKII and Bcl-2 family proteins from the complex and induces mitochondrial permeability transition (Reddy, 2013). There are three known isoforms of VDAC in mammals (De Pinto et al., 2010; Messina et al., 2012) and it has been described that VDAC2 mediates the translocation of GSK-3β to the mitochondria to trigger mPTP opening, as the knockdown of VDAC2, but not VDAC1 or VDAC3, attenuates both the mitochondrial translocation of GSK-3β and mPTP opening under stress conditions (Tanno et al., 2014).

**Figure 2:**
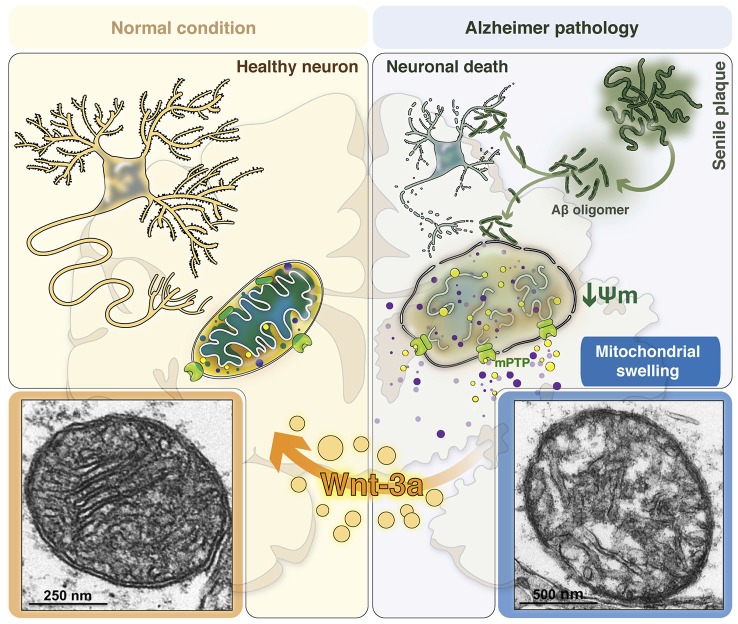
**Consequences of mitochondrial permeability transition in the AD brain: The role of canonical Wnt signaling in neuroprotection**. Mitochondria are structured organelles, which in normal or healthy conditions *(left panel)* present a conserved structure of both the inner and outer mitochondrial membranes. This organization allows the formation of the mitochondrial cristae, which are important since therein resides the electron transport chain to support cellular respiration and neuronal viability. Under AD conditions *(right panel)*, in the presence of Aβo, mitochondria undergo structural changes as a result of Aβ-induced mitochondrial membrane permeability. This phenomenon is triggered by the opening of the mitochondrial permeability transition pore (mPTP), which produces water and solute entry into the mitochondria, inducing increased mitochondrial volume and the loss of its structure, promotes mitochondrial membrane potential (mΔΨ) dissipation and the release of pro-apoptotic factors and calcium to the cytoplasm. These morphological changes that mitochondria undergo during membrane permeability are known as mitochondrial swelling, which finally generates the loss of mitochondrial function to activate neuronal cell death processes. Bottom panels show representative images obtained by electron microscopy of a healthy mitochondrion (left) with normal membrane and cristae structures and an AD mitochondrion (right) with disrupted membranes and cristae disorganization as a result of mitochondrial swelling induced by Aβo. Canonical Wnt signaling activation through the Wnt3a ligand prevents these morphological and structural changes, preserving the integrity and function of the mitochondria in the brain.

We have described that Wnt signaling activation increases mitochondrial HKII levels (Arrázola and Inestrosa, 2013), suggesting that the modulation of GSK-3β activity regulates the recruitment of HKII to the mitochondria in response to Wnt3a, reinforcing the idea that GSK-3β inhibition is a key event in mPTP inhibition. This allows us to propose that the Wnt signaling pathway controls the opening of the mPTP, and prevents mitochondrial membrane permeability and the subsequent collateral effects observed in AD (Figure 2).

### Non-Canonical: Controls Mitochondrial Dynamics

In the central nervous system, the non-canonical ligand Wnt5a plays a key role in the regulation of the postsynaptic compartment in neurons. It has been shown that Wnt5a induces the clustering of the postsynaptic density protein (PSD-95), through the activation of the Wnt/JNK pathway (Farías et al., 2009), increasing the traffic and retention of the GABA_A_ receptor at the neuronal cell surface, an effect mediated by CaMKII activation (Cuitino et al., 2010). These changes promote the generation of new spines through a mechanism that involves the activation of the Wnt/Ca^2+^ pathway (Varela-Nallar et al., 2010), and a redistribution of mitochondria to the spines, suggesting a new role for Wnt5a in the modulation of the mitochondrial network and function (Godoy et al., 2014).

**Figure 3:**
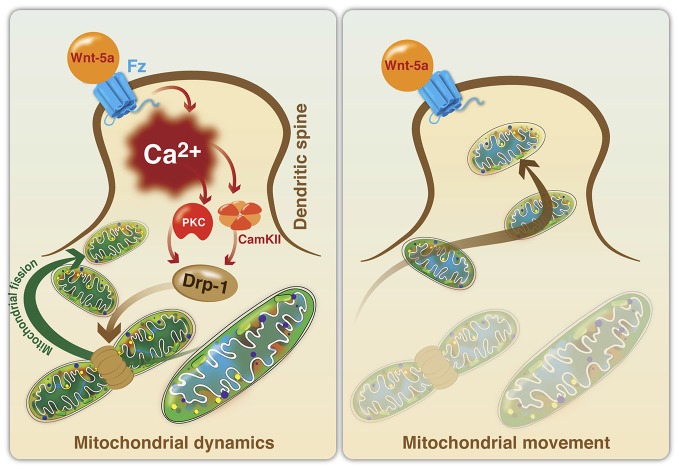
**Model of mitochondrial fission induced by the activation of the non-canonical Wnt signaling pathway in dendritic spines. *Mitochondrial dynamics***. Wnt5a ligand binds to its Fz receptor and activates the Wnt/Ca^2+^ signaling pathway to promote mitochondrial fission in the postsynaptic area. Wnt5a increases intracellular Ca^2+^, following the activation of kinases (PKC and CaMKII) and phosphatases (calcineurin), which in turn activates Drp1 through the regulation of its phosphorylation and dephosphorylation states. Drp1 translocates from the cytoplasm to the mitochondrial surface to induce mitochondrial fission. ***Mitochondrial movement***. The importance of the dendritic distribution of mitochondria in the generation of new spines and synaptic plasticity has been established. Once Drp1 is activated and induces mitochondrial fission, new mitochondria could mobilize to dendritic spines to support the energy demands associated with neural activity.

There is evidence suggesting that the Wnt signaling pathway participates in the regulation of mitochondrial dynamics and function. Recently, we established that Wnt5a can avoid mitochondrial loss in hippocampal neurons exposed to Aβ, through the regulation of mitochondrial dynamics *in vitro* (Silva-Alvarez et al., 2013). These effects are mediated by a Ca^2+^-dependent mechanism that involves the IP**3**R and the RyR from the ER. The relationship between mitochondrial fragmentation and ER-mediated calcium release has been previously reported in hippocampal neurons challenged with Aβo (Paula-Lima et al., 2011) and 4-CMC, a direct agonist of RyR, that reproduced the mitochondrial fragmentation effects observed with Aβo (Paula-Lima et al., 2011; Sanmartin et al., 2012). However, the mechanisms involved in the effects of Wnt5a on the neuroprotection against Aβ remain to be determined. The effects of Wnt5a on mitochondrial dynamics was recently described (Godoy et al., 2014). Wnt5a induced an increase of mitochondrial fission at the postsynaptic region, through the increase of intracellular and mitochondrial Ca^2+^ levels, and the regulation of Drp1 activity. In these conditions, the calcium increase activates several kinases, such as, PKC and calcineurin (Cereghetti et al., 2008; Qi et al., 2011) and our results suggest that these enzymes could activate Drp1 to control mitochondrial fission through Drp1 translocation from the cytoplasm to the OMM (Figure 3). The increase in mitochondrial number at the postsynaptic region suggests that Wnt5a might act as a physiological regulator of mitochondrial dynamics during synaptic function, collaborating with the energetic needs of neurons, as has been demonstrated for the activation of mitochondrial fission by Drp1 in dendritic spines (Li et al., 2004).

Considering that neurons are highly polarized and have an elevated demand of energy, mitochondrial distribution is critical for the proper function of neuronal cells. In non-mitotic cells, such as neurons, mitochondrial fission is particularly important since it allows to create new mitochondria and contributes to quality control by removing damaged mitochondria, facilitating apoptosis against high levels of cell stress (Youle and van der Bliek, 2012; Hoppins, 2014), such as in AD (Wang et al., 2008, 2009; Itoh et al., 2013; Silva-Alvarez et al., 2013). Interestingly, the increment of fragmented mitochondria in hippocampal neurons treated with Aβ dropped drastically after a short period of time, suggesting the elimination of mitochondria mass as part of the natural process of apoptosis against a major injury. This effect was abolished with Wnt5a, which in the presence of Aβ also caused an increase of fragmented mitochondria, and remained constant over time (Silva-Alvarez et al., 2013). A possible mechanism involved in Wnt5a neuroprotection in hippocampal neurons exposed to Aβ might be due to the increasing mitochondrial Ca^2+^ levels triggered by this ligand (Silva-Alvarez et al., 2013; Godoy et al., 2014). Neuronal mitochondria have a fundamental and strategic spatial distribution to buffer the increase of Ca^2+^. The presence of mitochondria in the vicinity of Ca^2+^ channels, such as NMDA receptors, allows mitochondrial Ca^2+^ incorporation and prevents mass propagation of cytosolic Ca^2+^ (Billups and Forsythe, 2002; Medler and Gleason, 2002). Clearly, increasing the buffering ability of mitochondria is essential for proper neuronal function, particularly in areas of high nervous activity. The stimulation of mitochondrial fission through Wnt5a would provide the energy requirements needed during nerve activity and, moreover, its effect on the buffer capacity of Ca^2+^ in mitochondria could protect neurons from the toxicity associated with the uncontrolled increase of intracellular Ca^2+^ levels produced during synaptic activity.

## Conclusion

Energy metabolism is a key process for the normal function of the body, mainly in the brain, an organ with high-energy dependency. In energy production, mitochondria play a central role, which is demonstrated by the diverse alterations of mitochondrial functions described in neurodegenerative diseases, such as AD, where deregulation of mitochondrial function is directly correlated with different pathological processes including decreased brain metabolism, alterations in mitochondrial structure, deregulation of regulatory enzyme activity, increased oxidative stress and increased levels of mitochondrial Aβ. Together, these changes have been proposed as markers of AD pathogenesis, with special focus on mitochondrial physiology. An interesting aspect of AD is the close relationship between the damage caused by the accumulation of amyloid plaques and mitochondrial dysfunction. Several studies have reported that the Aβ peptide can directly interact with the mitochondrial membrane promoting the opening of the mPTP, which triggers a deregulation in Ca^2+^ levels, leading to the dysfunction of several neuronal processes, irreversible failure of synaptic transmission and finally to neuronal cell death. In recent years, several groups have proposed that deregulation of the mitochondrial fission-fusion processes could play a key role in the initiation and progression of various neurodegenerative diseases, positioning itself as a possible common mechanism for the observed neuronal death in these pathologies and becoming an interesting therapeutic target. The search of strategies that could prevent the opening of the mPTP and the description of signaling pathways that can regulate mitochondrial dynamics, represent the first step in developing therapeutic strategies. In this regardor laboratory has described key evidence about the role of the Wnt signaling pathway in neuroprotection against Aβ and in the regulation of mitochondrial dynamics. Considering that Wnt signaling has been proposed as a critical pathway in neurodegeneration, this interaction could represent an interesting field of study in the search for drugs capable of reversing mitochondrial dysfunction and in this way avoid or revert the onset and progression of neurodegenerative diseases.

## Conflict of Interest Statement

The authors declare that the research was conducted in the absence of any commercial or financial relationships that could be construed as a potential conflict of interest.

## Abbreviations

Aβo: amyloid-beta oligomers
AD: Alzheimer’s disease
ANT: adenine nucleotide translocase
CMT: Charcot-Mary-Tooth disease
CypD: cyclophilin D
ER: endoplasmic reticulum
GSK-3β: glycogen synthase-3 beta
HKII: hexokinase II
IMM: inner mitochondrial membrane
IP_3_R: inositol triphosphate receptor
mΔΨ: mitochondrial membrane potential
MM: mitochondrial matrix
mPTP: mitochondrial permeability transition pore
OMM: outer mitochondrial membrane
PD: Parkinson disease
PSD-95: postsynaptic density protein 95
ROS: reactive oxygen species
RyR: ryanodine receptor
SNCA: α-synuclein
VDAC: voltage-dependent anion channel.
